# Urinary Pharmacokinetic and Pharmacodynamic Profiles of Fosfomycin against Extended-Spectrum β-Lactamase-Producing *Escherichia coli* with Canine Ex Vivo Modeling: A Pilot Study

**DOI:** 10.3390/antibiotics9050230

**Published:** 2020-05-05

**Authors:** Kazuki Harada, Takae Shimizu, Koji Kawaguchi, Takeshi Furuhashi, Genki Ishihara

**Affiliations:** 1 Department of Veterinary Internal Medicine, Tottori University, Tottori 680-8553, Japan; takae.shimizu@ani-com.com (T.S.); betty.0109.03@icloud.com (K.K.); 2 Anicom Specialty Medical Institute Inc., Kanagawa 231-0033, Japan; takeshi.furuhashi@ani-com.com (T.F.); genki.ishihara@ani-com.com (G.I.)

**Keywords:** dogs, urinary tract infection, fosfomycin, extended-spectrum β-lactamase-producing bacteria, ex vivo model

## Abstract

Fosfomycin is a candidate drug for extended-spectrum β-lactamase (ESBL)-producing bacteria, but its efficacy is yet to be investigated in dogs. This study investigated the urinary pharmacokinetic/pharmacodynamic (PK/PD) profile of fosfomycin orally administered at 80 mg/kg to six healthy dogs to assess its efficacy for canine urinary tract infections (UTIs) caused by ESBL-producing bacteria. Four strains of ESBL-producing *Escherichia coli* (ESBL-EC) characterized by fosfomycin minimum inhibitory concentrations (MICs) of 0.5, 1, 2, and 32 µg/mL were used. Urine samples for the measurement of urinary drug concentrations and urinary bactericidal titers (UBTs) were obtained after drug administration. The urinary concentrations (µg/mL, mean ± SE) were 1348.2 ± 163.5, 1191.6 ± 260.2, and 661.1 ± 190.4 at 0–4, 4–8, and 8–12 h, respectively, after drug administration. The mean urinary area under the curve during the test period (AUC_0–12_) of fosfomycin was estimated to be 12,803.8 µg·h/mL. The UBTs for all tested strains fluctuated closely with urine concentration during the test period (*r* = 0.944–1.000), and the area under the UBT-versus-time curve correlated with the urinary AUC/MIC of each strain (*r* = 0.991). According to the optimal urinary PK/PD target value, fosfomycin at 80 mg/kg twice daily may be suitable for the treatment of canine UTIs caused by ESBL-EC presenting MIC ≤ 128 µg/mL.

## 1. Introduction

Bacterial urinary tract infections (UTIs) are common infectious diseases in dogs. Most UTIs can be successfully managed with appropriate antibiotic treatment; however, bacterial resistance, as well as a compromised host immune system, can result in persistent and recurrent infections [1]. Although a wide range of Gram-negative and Gram-positive bacteria may be detected as the causative organisms, *Escherichia coli* is the most common bacterial cause of canine UTIs [2,3]. The prevalence of extended-spectrum β-lactamase (ESBL) in Enterobacteriaceae and, particularly, in *E. coli* isolated from companion animals with UTIs is of great concern worldwide [4,5]. Although ESBLs are usually involved in resistance to oxyimino-cephalosporins, penicillins, and narrow-spectrum cephalosporins, ESBL-producing bacteria are often also resistant to other classes of antimicrobials [6]. These multidrug-resistant phenotypes of ESBL-producing bacteria present major complications in the selection of adequate empirical therapy regimens [6].

Fosfomycin is a bactericidal antibiotic that interferes with cell wall synthesis in both Gram-positive and Gram-negative bacteria [7]. Although fosfomycin is an “old” antibiotic, having first been discovered in 1969 [8], it has recently garnered attention as a candidate drug for ESBL-producing bacteria [9,10,11]. In fact, we previously confirmed the excellent in vitro efficacy of several fosfomycins against ESBL-producing *E. coli* (ESBL-EC) isolates from both companion animals [12] and humans [13]. In these studies, fosfomycin emerged as a promising candidate antimicrobial for canine UTIs with ESBL-producing bacteria. Unfortunately, fosfomycin, though approved as a human drug, has not been approved for veterinary use in most countries, and thus knowledge on this drug is insufficient in veterinary medicine. Notably, the urinary pharmacokinetic/pharmacodynamic (PK/PD) profile and clinical breakpoint of fosfomycin are yet to be investigated in dogs.

In the present study, we used liquid chromatography–mass spectrometry (LC–MS) to investigate the urinary PK of fosfomycin in dogs. We also measured urinary bactericidal titers (UBTs) against ESBL-EC strains from canine UTIs to assess the urinary PD of the drug.

## 2. Results

### 2.1. Safety and Laboratory Test Results

No adverse effects were observed in any dogs during the test period. The results of the physical examination, complete blood count, and biochemical blood test showed no clinically relevant changes.

### 2.2. Urinary Concentration

**Figure 1:**
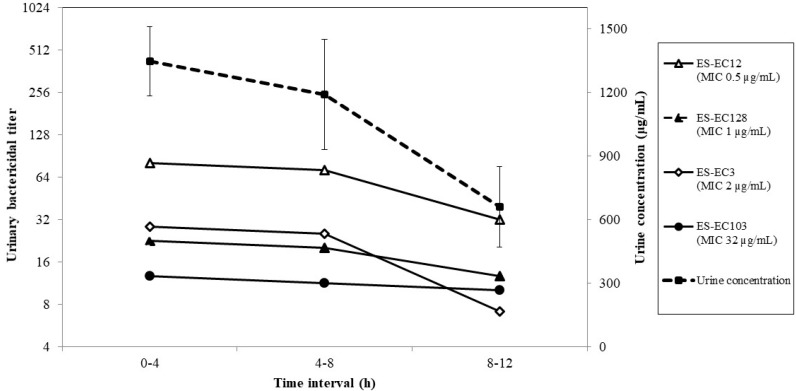
Urinary concentrations and urinary bactericidal titers (UBTs) of fosfomycin in dogs administered a dose of 80 mg/kg body weight (Mean ± SE, n = 6).

The LC–MS assay showed a minimum detection level of 0.5 µM (approximately 69.5 ng/mL) for fosfomycin. A correlation coefficient of 0.979 was given by the calibration curve. The urinary concentrations (mean ± SE) were 1348.2 ± 163.5 µg/mL at 0–4 h, 1191.6 ± 260.2 µg/mL at 4–8 h, and 661.1 ± 190.4 µg/mL at 8–12 h after administration (Figure 1). The *T*_1/2_ (mean ± SE) in urine was 10.03 ± 3.72 h. All urine samples collected prior to drug administration showed no detectable drug presence. Based on urinary pharmacokinetics, the mean urinary area under the curve during the test period (AUC_0–12_) of fosfomycin was estimated to be 12,803.8 µg·h/mL. The mean urinary AUC/minimum inhibitory concentration (MIC_0–12_), as well as the urinary Cmax/MIC and the urinary time above MIC (%TAM), were calculated for each tested strain, as shown in Table 1.

### 2.3. Urinary Bactericidal Titers

The fluctuations in median UBTs for each strain at different urinary concentrations are shown in Figure 1. A temporal change of the geometric means of fosfomycin UBTs for strains ES-EC12, ES-EC128, ES-EC3, and ES-EC103 correlated significantly with the urinary concentration (*r* = 0.999, 1.000, 0.996, and 0.944, respectively).

The median values of the area under the UBT-versus-time curve within 12 h after administration (AUBT_0–12_) for each tested strain are shown in Table 1. The correlation coefficient between AUBT_0–12_ and AUC/MIC_0–12_ and that between AUBT_0-12_ and C_max_/MIC were both 0.991 (*p* < 0.05).

## 3. Discussion

Although fosfomycin shows excellent in vitro antimicrobial activity against ESBL-EC isolated from companion animals [12], its efficacy for canine UTIs caused by these bacteria has not been previously assessed. To our knowledge, this is the first study investigating the urinary pharmacokinetics and pharmacodynamics of fosfomycin in dogs.

The urinary pharmacokinetic analysis of fosfomycin in this study demonstrated that fosfomycin is excreted at extremely high maximum concentrations in urine (1348.2 µg/mL). This is more than 100 times its concentration in blood (10.84 µg/mL) at the same dose [14]. In addition, the urinary half-life (*T*_1/2_) (10.03 h) of the drug was much longer than its plasma *T*_1/2_ (2.18 h) [14]. Similar findings were confirmed in the pharmacokinetic analysis of fosfomycin in humans [15,16,17]. In combination, these findings indicate significant differences between the blood and the urinary pharmacokinetics of fosfomycin, underpinning the superiority of the drug for the treatment of UTIs in dogs as well as humans.

The %TAM, C_max_/MIC, and AUC/MIC are regarded as representative PK/PD indexes indicating the clinical efficacy of antimicrobial drugs [18]. Although the optimal PK/PD index for the efficacy of fosfomycin has yet to be agreed upon, Zykov et al. [11] recently used a murine UTI model to confirm that AUC/MIC and C_max_/MIC were the indexes best correlated with in vivo activity of the drug. They also suggested that the optimal urinary AUC/MIC_0–72_ ratio is >600 (i.e., the optimal urinary AUC/MIC_0–12_ ratio is >100). Our data showed that the urinary AUC/MIC_0–12_ of fosfomycin is 12,803.8 in dogs administered 80 mg/kg of the drug, and thus a twice-daily administration of the drug at this dose would theoretically have in vivo efficacy for canine UTIs caused by ESBL-EC strains with MIC of ≤128 µg/mL. Also, the same regimen of fosfomycin would achieve the optimal urinary %TAM during the dosing interval for 100% of the examined ESBL-EC strains with MIC ≤128 µg/mL, determined as the cumulative percentage of time that the drug concentration exceeds by fourfold the MIC of the pathogen [18]. These findings suggest that a fosfomycin MIC ≤128 µg/mL is a reasonable clinical breakpoint for the treatment of canine UTI with 80 mg/kg twice daily when the optimal PK/PD index of the drug is either AUC/MIC or %TAM. This putative breakpoint is comparable to those for *E. coli* in human UTIs (i.e., ≤64 µg/mL for susceptible, 128 µg/mL for intermediate, and ≥256 µg/mL for resistant *E. coli* infections), according to the Clinical and Laboratory Standards Institute guidelines [19]. Recently, VanScoy et al. [20] proposed the %TAM as a preferred PK/PD index for fosfomycin efficacy for resistant bacterial subpopulations, based on results obtained by using an in vitro infection model. Thus, further consideration may be given to optimal PK/PD indexes and the breakpoint of fosfomycin for canine UTI.

Urinary drug concentration is indeed correlated with antibacterial activity in UTIs; however, the activity of antimicrobial drugs can be affected by the biological urine matrix [21]. Notably, the bactericidal activity of fosfomycin may be affected by glucose-6-phosphate, although this molecule is usually absent in urine [15]. UBTs can serve as PK/PD assessment parameters of antimicrobial agents in the urine [22]. Thus, we determined the UBTs of fosfomycin and urinary concentrations in dogs, after drug administration. Our results indicated that the UBTs of fosfomycin in all tested strains fluctuated closely in accordance with the urine concentration of the drug during the test period. In addition, the values of AUBT, a time integration of UBTs, greatly correlated with the urinary AUC/MIC and C_max_/MIC of each strain, although the correlation coefficient may not be robust because of the small data volume. These findings indicate that concentrations of fosfomycin in dog urine strongly reflect its bactericidal activity.

Previously, we reported that the values of MIC_50_ and MIC_90_ were 1 µg/mL and 4 µg/mL, respectively, for a large collection of ESBL-EC isolates from companion animals [12] and we also found that all the ESBL-EC strains of canine UTIs had MIC ≤32 µg/mL (data not shown). Therefore, the administration of fosfomycin at 80 mg/kg twice daily would have clinical efficacy for almost all cases of canine UTIs by ESBL-ECs, because the putative MIC breakpoint (i.e., ≤128 µg/mL) will usually exceed the MICs of even wild-type ESBL-EC strains causing canine UTIs. However, further clinical trials are needed to clarify the clinical efficacy of fosfomycin for canine UTIs caused by ESBL-EC.

## 4. Materials and Methods

### 4.1. Sampling of Urine from Dogs Treated with Fosfomycin

Six beagle dogs (four males and two females; mean age 6.9 ± 3.2 years, weight 11.3 ± 1.6 kg) were purchased from Kitayama Labes Co., Ltd. (Nagano, Japan). Prior to this study, all dogs underwent a physical examination, complete blood count, biochemical blood test, and urinalysis and were confirmed to be clinically healthy. A balloon catheter was placed in the urinary bladder of each dog to allow urine collection. Fosfomycin (Fosmicin^®^; Meiji Seika Pharma Co., Ltd., Tokyo, Japan) was orally administered at a dose of 80 mg/kg body weight, according to a previous study [14]. Whole urine was obtained via the catheter 4 h, 8 h, and 12 h after administration of the drug. This sampling schedule was set based on the recommended regimen of fosfomycin in dogs (i.e., every-12-hour administration) [14] and extremely slow transition of urine concentration [23]. The samples were sterilized using 0.22 µm-pore size filters (Starlab Scientific Co., Ltd., Shaanxi, China) and stored at −80 °C until analysis.

The animal experiments in this study were conducted under an ethics committee-approved protocol in accordance with the Tottori University Animal Use Committee (approval number: 15-T-46).

### 4.2. Measurement of Urine Fosfomycin Concentration with LC–MS

The solid phase extraction (SPE) method was used for pre-treatment of the urine samples. Briefly, 1 mL of urine sample was mixed with the same volume of 100% methanol and centrifuged at 21,000× *g* for 3 min. The supernatant was dried by a centrifugal vacuum concentrator (CC-105, TOMY SEIKO Co., Ltd., Tokyo, Japan) with an Ulvac DTU-20 (ULVAC KIKO, Inc., Miyazaki, Japan) connected to a cold trap (TU-500, TOMY SEIKO). The dried pellet was dissolved in 50 μl ultra-pure water and mixed with 1 mL methanol–chloroform (5:2) solution. The solution was vortex-mixed for 1 min and then centrifuged at 21,000× *g* for 3 min. The supernatant was dried using a centrifugal vacuum concentrator. The dried pellet was dissolved in 500 μl ultra-pure water and then loaded onto a Bond Elut Plexa SPE cartridge (Agilent Technologies Japan, Ltd., Tokyo, Japan). The flow-through fraction was collected by centrifugation, lyophilized, and dissolved in methanol–chloroform–water (5:2:1) extraction buffer. The solution was loaded onto an InertSep GC SPE cartridge (GL Sciences Inc., Tokyo, Japan). The flow-through fraction was collected by centrifugation and dried using a centrifugal vacuum concentrator. Finally, the pellet was dissolved in 50 μl of ultra-high-quality water.

LC–MS was carried out with a high-performance liquid chromatographer (Agilent 1260 Infinity, Agilent Technologies Japan, Ltd., Tokyo, Japan) equipped with a 6120 single-quadrupole mass spectrometer (Agilent Technologies Japan). An electro-spray ionization source interface operating in positive-ion mode was used for multiple reaction monitoring. The mass spectrum of fosfomycin is characterized by peaks at *m/z* 139. The compounds were separated on a 4.0 mm internal diameter × 50 mm length, 3 μm analytical column operated at 35 °C (InertSustainSwift C18; GL Sciences Inc., Tokyo, Japan). The mobile phase comprised 0.1% ammonia aqueous solution and 100% methanol, and the flow rate was 0.5 mL/min. The injection volume was 0.1 μL. Standard samples for the creation of a calibration curve were prepared at four concentrations (i.e., 0.5, 1, 5, and 10 mM) of fosfomycin aqueous solution.

### 4.3. Test Organisms

The four ESBL-EC strains (ES-EC12, ES-EC128, ES-EC3, and ES-EC103) were selected from strains causing canine UTIs in our collection [12]. The fosfomycin MICs and ESBL types of these tested strains are listed in Table 1. The strain ES-EC103 was characterized by the highest MIC of fosfomycin (32 µg/mL) among the strains causing canine UTIs in our collection.

### 4.4. Determination of Urinary Bactericidal Titer

The UBTs correspond to the maximal dilution titer of urine allowing bactericidal activity for each examined strain and were determined using each dog’s urine as previously described [22,24,25]. Each well of the microplates contained 100 μL of the logarithmic serial twofold dilution, ranging from 1:2 to 1:1024, acquired by mixing an equal volume of the urine sample obtained every 4 h after drug administration with the respective individual dog’s antimicrobial-free urine obtained prior to drug administration. Subsequently, the tested organisms were inoculated at a final concentration of approximately 5 × 10^5^ colony-forming units (CFU)/mL. The bacterial number of inoculations was estimated based on the turbidity of the inoculation and then quantified by the standard plate count method. Inoculated plates, plates containing antibiotic-free urine samples (control), and 10 serially diluted urine samples obtained at 4 h, 8 h, and 12 h after drug administration were prepared for each dog and then incubated at 35 °C for 18 h. The subcultured urine was transferred to antimicrobial-free agar and then incubated at 35 °C overnight. The number of grown colonies was used to determine the bactericidal endpoint. The UBT was defined as a ≥99.9% reduction of the initially inoculated colony counts. A UBT of 0 was defined as no bactericidal activity, and a UBT of 1 was assigned when only undiluted urine displayed bactericidal activity. UBTs were transformed into ordinal data and described with reciprocal numbers [22,24,25]. Simultaneously with the UBT determination, the absence of growth of the tested organisms was confirmed in all urine samples which had not been inoculated.

### 4.5. Statistical Analysis

*T*_1/2_ in urine was calculated by linear regression of the semi-logarithmic plot of urinary concentration versus the midpoint of the urine collection time [24]. The area under the UBT-versus-time curve within 12 h after administration (AUBT_0–12_) was calculated as the sum of the products of the reciprocal UBT values and the respective time intervals (h) for each test organism, to easily compare UBT data among the tested strains [22,24,25]. The urinary AUC_0-12_ was calculated with the trapezoidal rule by assuming that a drug concentration remains constant in the urinary bladder during each time interval [24]. The urinary AUC/MIC_0–12_ was calculated by dividing the urinary AUC_0–12_ by the MIC of the organism.

Pearson’s correlation coefficient (*r*) was calculated between mean urine concentration and geometric mean UBT and between mean urinary AUC/MIC_0–12_ and median AUBT_0–12_. A *p*-value <0.05 was considered significant for all analyses.

## 5. Conclusions

There are several limitations in this study. Firstly, this was a pilot study and hence examined only a small number of dogs. Thus, the present results might be somewhat biased. Secondly, the urinary pharmacokinetics of fosfomycin was considered based on the urine concentration at limited time points and thus may include uncertainty. Thirdly, only healthy experimental dogs were used in the study, and the urinary pharmacokinetics and pharmacodynamics of fosfomycin may differ in dogs with UTIs or household dogs. Nevertheless, we strongly believe that the present study provides fundamental knowledge on the clinical application of fosfomycin for ESBL-EC-related UTIs in dogs and also contributes to the effective use of fosfomycin in veterinary medicine.

## Figures and Tables

**Table 1:** Urinary pharmacokinetic/pharmacodynamic (PK/PD) indexes and the area under the UBT-versus-time curve within 12 h after administration (AUBT_0–12_) of fosfomycin for the four extended-spectrum β-lactamase-producing *Escherichia coli* (ESBL-EC) strains tested in this study. AUC/MIC_0–12_, area under the curve/minimum inhibitory concentration during the test period.

Strains	MIC (µg/mL) ^1^	ESBL Type ^1^	AUBT_0-12_	AUC/MIC_0-12_^2^	C_max_/MIC ^2^	Time above MIC_0-12_ (%) ^2^
ES-EC12	0.5	CTX-M-55	1248	25,607.6	2696.4	100
ES-EC128	1	CTX-M-2	578	12,803.8	1348.2	100
ES-EC3	2	CTX-M-27	224	6401.9	674.1	100
ES-EC103	32	CTX-M-14	96	400.12	42.1	100

^1^ These data were obtained from a previous study [12]. ^2^ These indexes were calculated based on urinary pharmacokinetics in this study.
